# Cardiac Stem Cell-Loaded Delivery Systems: A New Challenge for Myocardial Tissue Regeneration

**DOI:** 10.3390/ijms21207701

**Published:** 2020-10-18

**Authors:** Antonia Mancuso, Antonella Barone, Maria Chiara Cristiano, Eleonora Cianflone, Massimo Fresta, Donatella Paolino

**Affiliations:** 1 Department of Health Sciences, Magna Græcia University of Catanzaro, Loc. Germaneto, 88100 Catanzaro, Italy; antonia.mancuso@unicz.it (A.M.); fresta@unicz.it (M.F.); 2 Department of Experimental and Clinical Medicine, Magna Græcia University of Catanzaro, Loc. Germaneto, 88100 Catanzaro, Italy; barone@unicz.it (A.B.); mchiara.cristiano@unicz.it (M.C.C.); 3 Department of Medical and Surgical Sciences, Magna Græcia University of Catanzaro, Loc. Germaneto, 88100 Catanzaro, Italy; cianflone@unicz.it

**Keywords:** patches, injectable hydrogel, myocardial regeneration, nanomedicine, cardiac stem cell, tissue engineering

## Abstract

Cardiovascular disease (CVD) remains the leading cause of death in Western countries. Post-myocardial infarction heart failure can be considered a degenerative disease where myocyte loss outweighs any regenerative potential. In this scenario, regenerative biology and tissue engineering can provide effective solutions to repair the infarcted failing heart. The main strategies involve the use of stem and progenitor cells to regenerate/repair lost and dysfunctional tissue, administrated as a suspension or encapsulated in specific delivery systems. Several studies demonstrated that effectiveness of direct injection of cardiac stem cells (CSCs) is limited in humans by the hostile cardiac microenvironment and poor cell engraftment; therefore, the use of injectable hydrogel or pre-formed patches have been strongly advocated to obtain a better integration between delivered stem cells and host myocardial tissue. Several approaches were used to refine these types of constructs, trying to obtain an optimized functional scaffold. Despite the promising features of these stem cells’ delivery systems, few have reached the clinical practice. In this review, we summarize the advantages, and the novelty but also the current limitations of engineered patches and injectable hydrogels for tissue regenerative purposes, offering a perspective of how we believe tissue engineering should evolve to obtain the optimal delivery system applicable to the everyday clinical scenario.

## 1. Introduction

Nowadays, cardiovascular diseases (CVDs) represent some of the main causes of death in the industrialized world, especially for low- and middle-income countries [1]. Costs for primary life-saving procedures in case of acute clinical syndromes as myocardial infarction (MI) and chronic heart failures (CHFs) are gradually increasing while the medical management of the chronic sequalae of the acute syndromes remains mainly based on standard classic approaches such as β-adrenergic receptor blocking agents, angiotensin-converting enzyme (ACE) inhibitors and mineralocorticoid-receptor antagonists [2,3]. Indeed, the pharmacological approaches, which target the dysregulated signalling mechanisms determining the pathological cardiac remodelling after massive cardiomyocytes death after MI, haven’t practically changed for decades and no new therapies able to improve individuals’ life expectancy and the prognosis of patients affected by CHF are available. Thus, in the last years scientists have significantly invested in searching for novel affordable and efficacious therapies, in order to improve the long-term patients’ prognosis with chronic and complex cardiovascular diseases [4,5]. To this goal, regenerative medicine offers promising scenarios, overcoming in principle both the limitations of heart transplantation, the only effective cure for the chronic failing hearts, despite its notorious shortcomings such as low availability, blood-type compatibility and organ rejection [6], as well as the occurrence of pharmacological side effects leading to the lack of therapy efficacy [7]. In particular, to generate new vital myocardium, repairing the injured heart, several research groups focused their attention mainly on: (i) exogenous therapies, such the use of embryonic stem cells and induced pluripotent stem cells [8,9] or (ii) the endogenous regenerative potential of the adult heart, either by reprogramming in situ non-contractile cells into new cardiomyocytes (CMs) [10], or by exploiting the biology of the endogenous CSCs [11]. In particular the latter two methodologies have been implemented in order to repair the heart after injury as a safe, well tolerated and efficacious treatment for CVDs. Transplantation of autologous and allogenic CSCs have shown beneficial effects in different models of heart damage in both in small and large animals [12], and they have been also been tested in small phase I/II clinical trials in CVD patients [13]. However, cell transplantation may be limited by their insufficient survival rate in the diseased human heart [14], and by their low engraftment in the injured situ [15]. For these reasons, cell therapy applied to cardiac regeneration has significant room for improvement before establishing an economically affordable, widely available and efficacious clinical approach.

In this contest, nanotechnologies could help to reduce the current limitations associated to cell therapies. In particular, one of the most promising approaches is based on the in vitro cells’ loading into 3D-scaffolds, such as hydrogels or patches, to improve their engraftment in the injured site, reducing their clearance rate and increasing their differentiation potential [16,17,18,19].

Thus, in this review we analysed several proof of concept studies related to stem cell-based therapy as promising agents to treat heart injury. Considering their high tropism for the adult heart tissue, we focused our attention on endogenous CSCs as a powerful regenerative and reparative tool that has increasingly attracted the attention in the regenerative medicine field with the aim to use them in both gene therapy, stem cell therapy and tissue engineering [11]. To this end, we reviewed several applications of nanotechnologies in cardiovascular medicine involving the development of scaffold to deliver cells and several molecules to implement the effectiveness of the treatment, in the myocardial tissue.

Looking out to the next future, the pre-loading of CSCs in 3D-scaffolds before their implantation in a heart injury or the delivery into in-situ forming scaffold could be a promising new solution as well as the re-solution of many limitations associated with current stem/progenitor cell transplantation approaching, eventually impacting the efficacy of the CVDs’ management.

### 1.1. Adult Heart Biology: From a Static to a Dynamic View

Historically the adult heart has been considered a post-mitotic organ, lacking regenerative capabilities when referring to the contractile mass, constituted by CMs, with no reproducible evidence showing that new CMs were formed in the adult mammalian myocardium. Indeed, it has been known since more than 4 decades that shortly after birth, CMs undergo terminal differentiation becoming incapable of further replications [20,21,22]. Based on this CM-centred “static view”, it has been a dogma for cardiovascular biology and medicine until the beginning of the new millennium that CMs were not replaced for the whole lifetime of mammalian species, including humans, in response to injury or as consequence of aging and that necrotic/apoptotic CM death ensued in pathological cardiac remodelling mainly characterized by the CM hypertrophic process [23]. Thus, on the basis of this dogma, it became clear that any regenerative approach to the adult heart should come from exogenous sources, through the transplantation of exogenous regenerative cells or imperative, or that to obtain endogenous cardiac repair it was necessary to genetically manipulate the adult CMs, in order to bring them back into cell cycle, to re-activate their mitotic capability [24]. Unfortunately, the latter approach, mainly leading to an increment of CM polyploidy or death, was found not efficacious both in vivo and in vitro [25,26]. Moreover, the re-activation of mitotic cycle in CMs brought an increase of neoplastic risk in the heart, an organ classically known for the its very low incidence of malign tumor formation [22,27].

Nowadays, the above described old paradigm is considered practically obsolete because several burgeoning pieces of evidence that emerged in the early 2000s have been instrumental to the development of the nascent new “dynamic view” of adult cardiac cell turnover as well of the renewal potential of the adult heart. Indeed, several studies have shown a continuous formation of new CMs in the adult myocardium of the heart of mammalian species including mice, rats, dogs, pigs and humans [13,28,29,30].

Nevertheless, the current available technologies have been inadequate so far to correctly and directly quantify the number of new formed CMs during adulthood and after injury, so that the estimation about their turnover rate is not yet widely accepted and it remains questionable because it is not based on direct experimental evidences but mainly estimated by mathematical extrapolations [13,28,31]. Despite the current available evidences disagree on CM turnover rate, it is generally accepted that the innate intrinsic myocardial self-renewal potential is not sufficient to replace all the CMs lost by MI and during CHF. Thus, this evidence pushed a call to develop new experimental protocols, procedures and studies to define safe and efficacious therapies to counteract CM loss by heart injuries and to finally and correctly quantify the ability of adult mammalian heart to produce new CMs both in physiological and pathological conditions [22,23,32]. Surely, the overall goal of this significant ongoing scientific effort is to define the origin of all the regenerative events in the adult heart producing new myocardial cells, in order to exploit this new knowledge towards the robust formation of newly-generated CMs after damage.

The discovery that the adult heart possesses a pool of endogenous CSCs [33], opened the era of tissue-specific stem cell-based therapy in the cardiovascular field giving to the heart “the chance of re-birth” after injury. The big challenge however remains to understand how to better activate the intrinsic regenerative potential of these cells in order to give them the possibility to re-build the damage heart. To reach this ambitious goal it is mandatory to fully understand the whole biology of adult CSCs.

### 1.2. The Role of Lin^−^/c-kit^+^ Cardiac Stem Cells

With the aim of ensuring an efficacious reparative therapy after heart injury, in the last twenty years several approaches based on transplantation of cardiac stem cells [34], cardiosphere-derived cells [35], and in situ CSC activation [36], have been tested. In particular, considering their high tropism for the myocardial tissue, CSCs have been largely employed in regenerative medicine. Initially CSCs were identified in rodents by the expression of a biomarker known as c-kit (CD117 or SCF-R), a tyrosine kinase receptor [33]. These progenitor cells expressing c-kit were also positive for the stem cell antigen 1 (sca-1) and MDR1 [11,33], markers already reported to be expressed in several regenerative cell lineages as in the case of the hematopoietic system [37]. Nevertheless, the isolation of the whole CM-depleted interstitial cardiac cells from the adult heart using c-kit alone as surface markers identify an heterogenous cell population mainly composed of already committed cells [38]. Thus, it was essential to identify other markers that, together with c-kit, could better delineate the phenotype of this endogenous cardiac cell population with stem and progenitor properties. Accordingly, it has been subsequently shown that the lone c-kit expression in a cardiac cell population is not sufficient to identify the true CSCs in the adult heart [34,39,40]. The population of c-kit^+^ cells in the adult heart is indeed mainly composed of partially committed or fully committed hematopoietic and endothelial cells expressing CD45 and CD31 as surface markers [34,39,40]. A negative sorting for CD45/CD31 is necessary to eliminate form the c-kit^+^ cardiac cells the vast majority of lineage-committed cells [34,39,40]. These cells are positive for the expression of stemness markers such as SCA-1, MDR-1, Abcg2 and CD166 and when cloned through single-cell-deposition they can be propagated for more than 65 passages, maintaining at any passage the same phenotype and the same expression of stemness genes such as Bmi-1, Gata4, Mef-2c, Nkx2.5 [34,40]. When cultured in suspension these cloned cells are able to form pseudo embryoid bodies, called cardiospheres, a typical feature of multipotency [34]. Cloned CSCs exhibit significant cardiac tissue regenerative capacity giving rise to a minimum of three different cardiogenic cell lineages (myocytes, smooth muscle and endothelial cells) both in vitro and in vivo [5,11,23,34,39,41,42,43,44]. In particular, it has been previously reported that CSCs display a high tropism for damaged heart. Indeed, they are able to home to the injured site after systemic administration where they are able to differentiate in cardiac cells, including the CMs [41]. After transplantation, this cell population is able to produce a paracrine effect within the heart by secreting several growth factors, such as IGF-1, TGF-β1 superfamily, neuregulin-1, periostin that strongly activate the endogenous pool of CSCs, improving both their cardiomyogenesis and vascular differentiation rate [11,45]. Indeed, it has been demonstrated a paracrine feedback loop among CSC, resulting in a more intense and fully autologous regenerative response [11,13]. On the basis of this paracrine effects, it was postulated and then proved the regenerative potential of allogeneic CSCs, which is in perspective an affordable, widely available and efficacious regenerative approach [11]. Despite the promising results obtained by the different studies summarized above, recent data from genetic fate map murine models claiming to faithfully follow the cardiac muscle progeny of any regenerative cardiac cells expressing c-kit or Sca-1 have questioned the existence and the role of endogenous CSCs [46,47,48,49,50]. Modified mouse models have used the technology of DNA recombinases for site specific recombination, which is a gold standard for stem cell biology in vivo [51]. Unfortunately, all these practically all these genetically modified mice carried a recombinase insertion which created a null allele of the targeted gene, that is c-kit or Sca-1. The latter had the consequences of creating a haploinsufficient gene with significant negative impact on the labeling of the targeted cells and their regenerative potential [23,39,52]. Even when the recombinase was inserted in the targeted gene (c-kit or Sca-1) without creating a null allele, the recombination efficiency was not proved to be sufficient to recombine the endogenous CSCs [48]. Therefore, the data arising from these genetic fate map models are inadequate to quantitatively and qualitatively follow the cardiomyogenic progeny of endogenous CSCs either during physiologic aging or in response to injury [43,53].New mouse models are necessary to finally answer the outstanding question of correct quantification of new cardiomyocyte formation by CSCs in the adult life.

On the other hand, despite the incontrovertible beneficial effects observed in patients with chronic heart failure using autologous and allogenic CSCs [54] and in patients with refractory angina using endothelial progenitor cells [55], clinical trials for myocardial regeneration remain still debated and controversial because the results are modest [56]. CSCs transplantation, as tested so far in humans, is not sufficient to promote an effective cardiac regeneration [57], and the low survival rate of transplanted cells further limits the therapeutic efficacy of cell therapy [57], coupled with the fact that CSCs could be rapidly removed from the body shortly after systemic injection. For all these reasons, the regenerative medicine field is actively working to find a way to deliver cells specifically to the damaged site in order to ameliorate their survival and then their engraftment.

## 2. Cardiac Tissue Regeneration

**Figure 1:**
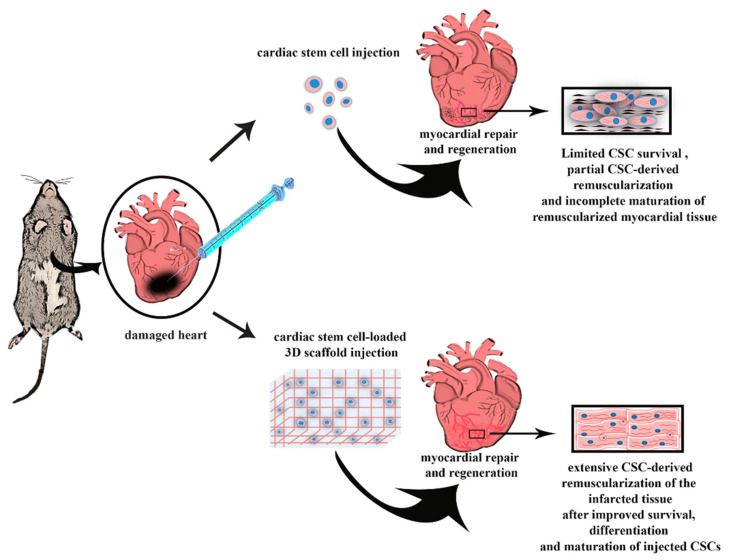
The cartoon depicts how a desirable delivery system should improve the efficacy of cardiac stem cell (CSC) injection to efficiently regenerate the infarcted heart. Indeed, classical CSC injections (either through direct myocardial delivery or through systemic administrations) obtain myocardial regeneration after myocardial infarction (MI) but the regenerated tissue is mainly located to the border zone when injected through the systemic circulation and when injected directly in the infarct zone the regenerated tissue is mainly composed by immature newly-formed cardiomyocytes derived by the differentiation of injected CSCs. A desirable engineered 3D delivery system (either a patch or a hydrogel, see text below) should sustain an increased survival of the injected CSCs improving their engraftment, and favoring their differentiation into fully functional and mature cardiomyocytes, obtaining a full cardiac regeneration of the infarcted myocardial tissue recovering heart normal function.

The potential role played by stem and progenitor cells and their efficacy in treating heart damage post myocardial infarction (MI) have already been widely described by scientific literature [54]. The investigation of cardiac cell therapy for cardiovascular disease management has been the aim of several preclinical and clinical studies [12,52], which have certainly demonstrated the importance of exploiting stem and progenitor cells in the treatment of heart diseases, but at the same time they have opened the scenario to the need of stem cells delivery systems. Indeed, the prevalent mechanism of action of cardiac cell therapy in humans has been associated with their ‘paracrine’ function [12,54,57]. Once delivered to the myocardium cells do not engraft on the long term but they reside enough to activate cell-to-cell connections, which mainly depend on the release by the transplanted cells of several reparative and regenerative factors. The latter foster endogenous myocardial activation, characterized by a decreased cell death and hypertrophy, an increase in cell survival and function of pre-existing cells while also improving neo-angiogenesis and in part neo-myogenesis [12,54,57]. In fact, it has been estimated that more than 90% of injected cells as suspension do not engraft in cardiac site and are easily lost after injection, leading to treatment failure [58]. Moreover, the cell-injection therapy could be effective only if carried out at the beginning of the acute damage, but at the same time the cells would be exposed to a very hostile environment, due to high immune response and high inflammatory process that occur in the infarcted area soon after insult, thus compromising the viability of injected cells [59,60]. So, a suitable delivery system appears necessary to increase the engraftment of the cells in the heart microenvironment, thus fostering their regenerative potential, while improving their survival upon transplantation (Figure 1).

To date, four main strategies have been investigated for cardiac tissue regeneration. In particular we can distinguish strategies involving cells or acellular materials: direct cell injection; the implantation of cardiac scaffolds; the injection of acellular materials able to trigger endogenous repair mechanisms; the use of biomimetic materials for valve replacement [61].

In regards with the implantation or injection of biomaterials alone as acellular materials, this strategy can be a temporary solution to reduce adverse post-MI tissue pathologic remodeling; but this procedure does not provide a long-term solution for tissue regeneration because the large number of cardiomyocytes lost during an ischemic heart attack is not adequately replaced. So, up-to-date cutting-edge nanotechnology aims to associate the use of biomaterials, as patches or injectable hydrogels, with the incorporation of CSCs. The use of these technologies for cardiac tissue regeneration is potentially advantageous because they could obtain a better engraftment of delivered stem cells, but to date several limits and critical issues remain to be resolved (Table 1) [58,59,60,62,63,64,65,66,67,68,69,70].

The main pending issues, concerning the use of biomaterials as patches or injectable hydrogels for cardiac regeneration are related to the complexity of heart microenvironment and pathophysiology following MI. Moreover, certain specific properties are necessary deliver stem cells using biomaterials as detailed below.

First of all, biomaterials need to be biocompatible and biodegradable; the latter means that they must perfectly adapt to the host tissue and protect the encapsulated cellular material avoiding the triggering of immune responses; moreover, once implanted, the biomaterial should possibly undergo the enzymatic degradation in a time-controlled manner and the biodegradation products must comply with the same biocompatibility and biodegradability requirements as the employed biomaterial [59]. While natural materials have rapid and well-defined degradation kinetics in vitro and in vivo [71], using synthetic materials it is possible to obtain a modulation of degradation rates, modifying their chemical structures. The biodegradation rate and the residence time of the biomaterial in the application or injection site are important factors to be evaluated. Indeed, a patch or a hydrogel should remain in the site of application for the time needed to favor the migration of the implanted cells and the replacement of dead cells which occurs following a heart attack. At the same time, the constructs should not remain too long at the administration site, because otherwise they would prevent tissue regeneration acting as a physical obstacle to tissue growth and remodeling. The carefully design of patches or hydrogels is essential for a successful implant treatment. A significant degree of attention is required by the intervention timing, i.e., the time elapsed between the heart attack and the intervention [59]. In particular, compared to the direct injection of cells that is needed soon after the heart attack, the application of the biomaterial is recommended within hours or 1–3 days after MI, so that it can reduce the formation of scar tissue, maintain the cardiac contractile function and reduce pathological remodeling [72]. The use of the right treatment and intervention time is a fundamental step and still remains a challenge. In any case this aspect is extremely controversial because a therapeutic administration at the wrong time could exacerbate the myocardial damage.

## 3. Epicardial Implanted Cardiac Patches

Cardiac patches are designed to replace “a piece of heart that is no longer functioning”. When designing a patch as delivery system for CSCs (and their progeny) and/or biological components, the right choice of the stem cells population is fundamental, but also and above all, the choice of materials to be used, from the technological-formulative point of view, is equally important. Several materials are available for tissue engineering, each one with their pros and cons; in any case, the selected material should create a fully integrated patch, able to support biological activity, avoid adverse host immune response, and support the contraction, shear stress and other dynamic forces of the heart [60]. Generally, the materials used for cardiac patches can be classified in natural and synthetic materials [73]. Alginate [74], collagen [75], Matrigel [76,77] and above all decellularized extracellular matrix (dECM) [78,79] are the most used natural materials for the preparation of cardiac patches. dECM is widely used for patches synthesis and for stem cell delivery. It is obtained by the removal of cells from a tissue or an organ, leaving the complex mixture of structural and functional proteins, glycosaminoglycans, proteoglycan such as native tissue [80,81]. Moreover, dECM is characterized by a good biodegradability; the in vivo and in vitro disruption occurs by specific enzymes secreted by cells in a specific time range (days to 1 week) and its degradation products are biocompatible and biodegradable, as required by tissue engineering. The main advantage of using dECM for the production of patches is the high affinity between the embedded and endogenous ECM and the progressive replacement of biodegraded dECM with new endogenous ECM components produced by the endogenous cells. Consequently, the dECM is able to induce a remodeling of the native tissue [71]. The importance of biocompatibility, especially for natural materials, is mainly related to a reduction of immune reactions, occurring as a result of the introduction of non-self-materials in the human body. Unfortunately, the issue of triggering the immune response following the cell-injection or the administration of cells-loaded scaffolds still remains one of the main limits to be elucidated and overcome for engineered combinations of biomaterial with stem cells and their differentiated progeny for remuscularization purposes, because in most cases it would lead to biograft rejection. When allogenic stem cells are injected into cardiac tissue or when they are applied to the myocardium using a scaffold, if the injected cells or the engineered biomaterial-cells construct are indeed used to obtain direct myocardial regeneration, their engraftment and their ensuing cardiac differentiation within the host myocardium would activate the host-derived reactive T lymphocytes, which would recognize non-self-human leukocyte antigens (HLAs) on the surface of transplanted donor cells [82]. Some studies on allogeneic stem cells implantation, suggested immunosuppression to preserve cell viability [83], and to favor the integration of the construct thus avoiding its rejection [84], but of course this is not without risk. Contrariwise, other research groups, proposed the modification of natural biomaterials to refine their features obtaining new biomaterial integrated by natural components of tissues, including progenitor cells, growth factors and, ECM structural components [85].

Another important feature to be evaluated is the thickness of the obtained construct, closely related to the mechanical support provided by the graft to the damaged heart and implanted cells. An experimental study has demonstrated that patches characterized by a thickness less than 400 micrometers ensured the maintenance of a certain viability of the implanted cells, probably due to a simpler vascularization network that is established between host heart and patch. On the contrary, for thickness values over 400 micrometers, a poor cell survival was observed and, according to the authors, this was probably related to the limited oxygen diffusion [86]. To overcome this limit, an in vitro vascularization of the patch before implantation has been suggested to both promote the nutritional support to the cells and increase the possibility of using a thicker patch that can give more mechanical support. With regards to mechanical strength, it was estimated that cardiac stiffness during diastole and systole is 20 kPa and 500 kPa, respectively [59]. Natural materials, derived from in vivo sources, such as decellularized ECM, are able to mimic cardiac microenvironmental architecture but they do not have sufficient mechanical strength to support the cardiac diastole and systole phases because they can achieve a stiffness of only a few dozen of Pa [73]. Wall and co-workers have demonstrated that the use of pre-formed patches characterized by an elastic modulus equal to a few tens of kPa is more effective than the use of patches with values of tens of Pa, because an higher stiffness when compared to cardiac muscle provides a major attenuation in wall stress [87]. An increased stiffness can be obtained modifying and carrying out crosslinking starting from natural biomaterial. For example, the crosslinking of dECM with biocompatible polysaccharides, as chitosan and alginate, has been recently proposed as a strategy to improve its mechanical strength [88], but this approach needs to be further investigated with in vivo studies to probe its efficacy. To overcome the issue of limited stiffness of natural biomaterial-based patches, synthetic materials could be exploited. The most commonly used are polycaprolactone, polyurethane, poly-(L-lactic) acid and poly(glycerol sebacate) (PGS) [59]. These synthetic materials allow for the production of a scaffold characterized by better defined physical and mechanical properties, obtaining an ad-hoc system with specific features. An example of synthetic material used to obtain a more appropriate mechanical features of patches is the elastomer PGS; thanks to its elastic nature and its Young’s modulus (considered excellent if between 22 and 50 kPa) [89], PGS is able to mimic the mechanically dynamic cardiac environment [90], as also demonstrated by Chen and co-workers. Authors affirmed that cellularized PGS cardiac patches did not alter systolic and diastolic function within two weeks after their implantation on the left ventricle of adult rats [91].

The choice of materials to use is not easy; as an example, the synthetic materials are able to provide the correct mechanical support to the infarcted heart but at the same time could affect the pumping function of the heart and the propagation of the electrical signal with consequent arrhythmias [73]. Unfortunately, arrhythmias represent a very common problem for standard cell therapy, and ventricular arrythmias are life-threatening complications, and the use of cellularized or acellularized cardiac patches does not exclude arrhythmias development [92], especially when applied to the clinical arena. Arrhythmia is characterized by alterations on the propagation of the electrical signals, and following the administration of the graft, unsynchronized electrophysiological signal transmissions, between the graft and host myocardium, can more often occur [93]. The possible explanations for the onset of arrhythmias in presence of cellularized cardiac patches are different: the great distance that the electrical signal has to cross; the presence of fibrotic scars [94]; and the presence of cells included in patch. In the case of cellularized patches, the inconvenience of arrhythmias could be linked not directly to the patch, but to the cells seeded on it. Implantation of mesenchymal stem cells has shown to induce arrhythmias [95], influencing the speeds of cardiac electrophysiological signals. In particular, implanted pluripotent stem cells-derived cardiomyocytes may alter the cardiac contractile activity [96]. Due to their similarity to neonatal cardiomyocytes [97], stem-cell derived myocytes included in the graft could act as pacemaker cells, inducing electric instability and consequently causing arrhythmias [92]. In these cases, the use of synthetic materials for cardiac patches permits to modify and optimize some aspects owing to the reduction of arrhythmia risk and a better control of cardiac tissue contraction after graft implantation. Feiner and co-workers have proposed an innovative cardiac patch equipped by gold electrodes [98]. The authors have conceived and produced an engineered and sophisticate cardiac patch characterized by an electronic network, maintaining the required porosity and small thickness. This microelectronic cardiac patch (microECP) was able to remotely activate the growing tissue and to control electric signal propagations. In this way, abnormal heartbeats could be prevented.

Studying and defining the correlation between the implantation of cardiac patches and the initiation of arrhythmias is not privy of bottlenecks, due to the different response of animal models used in in vivo experiments. This makes difficult the classical ‘bench-to-bedside’ translation, which is the passage from the animal model to the human patient. For this reason, several research groups, including Chong and co-workers [99], analyzed how animals respond to the transplantation of human embryonic stem cell-derived cardiomyocytes (hESC-CM), as a function of their size. In their studies, hESC-CM transplantation induced arrhythmias in monkey heart but not in smaller animals. The authors explained this dissimilar response as a consequence of the different animal heart size and rate. Substantially, the large hearts of no-human primate compared to that of other smaller animals, such as mice and rats, would require the delivery of a greater number of cells and the implant of a bigger patch; consequently, a slow conduction could occur in a graft having certain dimensions. Moreover, no-human primates present a lower heart rate (100–130 beats/min) with respect to mice and rats (600 and 400 beats/min respectively); as for the latter, the authors suggested that a faster heartbeat is less affected by graft automaticity, developing fewer arrhythmias. If this concept were further confirmed, it could explain the difficult clinical translation in humans since the human heart is larger and with a slower heartbeat than monkeys.

Independently of the selected material, the cardiac patches must guarantee the electrophysiological communication above mentioned, but also the physical and biochemical continuity and nutrient supply for the seeded cells and cardiac tissue [68]. Once implanted, engineered cardiac graft find a hostile ischemic microenvironment, which leads to a prominent cell death; the lack of blood supply after transplantation still further reduces the number of viable cells [100]. Vascularization of engineered tissues in vitro and in vivo remains another key problem in the translation of engineered tissues to clinical practice [101]. To reduce these problems, thicker multiple-cell layered cardiac patches with integrated engineered vessels or a primitive vascular network able to anastomosing with host vessels have been introduced [65]. In this way, the oxygen diffusion was improved and a good nutrient apport to the encapsulated cells was guaranteed [102,103].

In a recent study, Su and collaborators showed an example of a cardiac patch enriched with micro-engineered blood vessels tested on rats with acute MI [104]. Biomimetic microvessels, characterized by endothelial cells of the umbilical veins (HUVECs), were obtained by hydrodynamic microfluidics and were integrated into a specific patch made of fibrin gel containing human CSCs. Microvessels were able to provide nourishment to the stem cells and favor their efficacy of action. Results showed an increasing formation of cardiomyocytes in the peri-infarcted region and greater neovascularization in the infarcted region when compared with rats treated with the conventional patch containing only stem cells. Recently, also Quian and co-workers tried to reproduce the typical ordered physiological microvascular network of the myocardium [102]. Authors directed the alignment of microvascular network using sheets of decellularized human dermal fibroblast and performed a co-culture of human mesenchymal stem cells and endothelial cells. Their engineered construct influenced the vascular network development thanks to the enhanced vascular alignment, angiogenic growth factor secretion and ECM remodeling. A dense vascular network was obtained, so the authors hypothesized that loading the sheets with an excellent pre-vascularized 3D tissue could be obtained. All of these studies, along with others [105,106], confirmed that a patch containing preformed micro-vessels can be rapidly perfused once implanted, due to its integration with the in vivo host vascular network. This process induces the regrowth and remodeling of the heart tissue, reducing the harmful effects after MI.

To improve angiogenesis after patch implantation, growth factors, as vascular endothelial growth factor (VEGF), were recently proposed to enrich the cardiac patch. Miyagi et al., achieved significant results both in vitro and in vivo, using a seeded cells-patch prepared with immobilized VEGF for replacing a full right ventricular wall defect in rats. In vivo, the patches enriched with VEGF induced a greater blood vessel density than patches without supplement already after 7 days; after 28 days from implantation, the VEGF-patches were significantly thicker than control. The authors highlighted that the induced angiogenesis contributed to improve cell survival and tissue formation [101].

Pre-formed patches containing stem cells with or without any nutrients are characterized by some limits, but as described, the tissue engineering continues to propose solutions to exploit the benefits of cardiac patches. The big challenge for pre-formed patches is their in vivo implantation. To date, most cardiac patches require an open-chest surgery, and the invasiveness of this procedure leads to various problems that can lead to the failure of the entire implant operation [107]. An open-heart surgery is a great risk for the patient already debilitated by a heart attack; in fact it increases the risk of infections and inflammation, it exposes the patient to the possibility of forming pericardial adhesions, it requires a long hospitalization, and it also increases morbidity and mortality [66]. To date, the most plausible alternative to these patches which, despite being valid, involve risks for patients due to their method of implantation, seems to be the use of injectable in situ forming hydrogels.

## 4. Injectable in Situ-Forming Hydrogel

Hydrogel are water-swollen polymeric structures, characterized by more or less weak interaction of the constituent polymers. They have been the focus of several research studies since the mid-1930s and they have gained considerable interest over time due to their potential applicability in the biomedical field [108].

Injectable hydrogel application in cardiac tissue engineering is due to their viscoelastic nature and their chemical-physical versatility, making them suitable for cardiac regeneration [109]. Many features required by pre-formed patches for safe and effective stem cell delivery also concern hydrogels. Properties such as electrical conductivity, biodegradability, biocompatibility and adequate mechanical strength are features that a hydrogel must necessarily possess to ensure its correct implantation and good cell viability.

A specific feature of injectable hydrogels is their ability to absorb water from the environment that lead to an increase in their volume and make them similar to soft tissue. The swelled hydrogel is characterized by a certain porosity that permits to retain any incorporated cells and several nutrients or active molecules, useful for preserving the vitality of cells from the cardiac microenvironment during inflammation [110].

A correct consideration of the cardiac microenvironment, which is derived as a result of MI, could be helpful to intervene inserting bioactive compounds, such as antioxidants, which aid cells seeded into the injectable hydrogel to endure the hostile microenvironment. The latter is the case of a recent work performed by Hao and co-workers that prepared a fullerenol/alginate hydrogel, with a gelation time completed within 5–10 min. This hydrogel showed a great ability to suppress the high stress damage caused by reactive oxygen species (ROS) in a rat model with MI, due to the presence of fullerenol. In this way it allowed an excellent retention and survival of the encapsulated brown adipose-derived stem cells (BADSC), also leading to a reduction of the infarct size, and the increase in wall thickness and cardiac function [111].

Several preparation methods help to obtain a homogeneous formation of hydrogel containing stem cells [112], but the use of cell suspension included in a gelling precursor solution seems to be the most effective. In fact, a homogenous distribution of stem cells into hydrogel occurs during crosslinking between gelling polymers of network, trapping the stem cells included in solution in their meshes. The number of cells suspended in this delivery system is variable and it is strictly related to the type of cells used and the type of materials that define the structure [64].

Injectable hydrogels seem to be advantageous if compared to the use of patches for cardiac tissue repair, because they can be administrated by a minimally invasive procedure (like percutaneous coronary interventions or percutaneous cardiac catheterization in general, which ensures a better patient compliance while also better protecting the encapsulated cells from the shear forces of the injection itself [113]. However, to guarantee a safe and minimally invasive injection, hydrogel must be able to pass through a fine gauge needle (~27G) [59]. To do this, the ideal hydrogel should be administered in liquid form and undergo a sol-gel transition in situ following specific stimuli. The in situ gelling would also allow the hydrogel in liquid form to stratify on the damaged site and once jellified to deform following the dynamic myocardial behavior and interact with endogenous ECM, facilitating a better interaction with the host tissue. In this way the in situ-forming hydrogel should provide mechanical support, improve left ventricle function through an improved remodeling of the damaged tissue and obtain an increase in wall thickness attenuating wall stress [114].

If in situ gelling is the most advantageous practice for tissue engineering, in some cases, gelling can begin outside the body and be completed inside in a time long enough to allow the passage through the needle but not to permit the dilution into the body fluids, once injected. Chemical crosslinking (Michael addition reaction) or ionic crosslinking can be exploited to obtain these pre-gel solutions [115]. For example, Chow and co-workers recently used a thiol-Michael addition reaction to realize polyethylene glycol (PEG) hydrogels, adding PEG dithiol to 4-arm PEG acrylate. According to the authors, the optimum gelation time is around 180 s to permit the pre-gel administration through catheter with completed crosslinking once in situ. For this reason, the authors have chosen the formulation made up of 10% PEG for the in vivo studies, confirming that the rate of crosslinking process is dependent from polymer concentration. The selected formulation was injected in a rat model of MI, demonstrating that it was able to increase thickness of the infarcted region and cardiac functions [116].

Light-inducible photo-crosslinking is another procedure to induce in situ jellification of a liquid polymeric solution. This mechanism induces a very quick gelling reaction following UV-light exposure that stimulates the crosslink formation between polymers. The obtained gels include the required technological features of high elasticity, mechanical support, easy administration and very short gelation time in situ, but in some cases, it could negatively impact on human body and on its normal functions. In detail, UV light can generate reactive oxygen species, inducing a probable endogenous oxidative damage to DNA, and so it can cause accelerated tissue aging and cancer, at level of the irradiated zone. Accordingly, although their demonstrated efficacy in the induction of vascular networks formation and tissue healing [117], in situ UV crosslinking of prepolymers, such as gelatin methacryloyl, may negatively impact the normal function of the myocardium. Noshadi and co-workers overcame this issue realizing a gelatin based injectable hydrogel functionalized with Eosin γ that operates as photo-initiator. This engineered gelatin solution undergoes photo-polymerization if exposed to visible light, that is less harmful for human body, and the obtained in situ gel was characterized by good porosity, swelling behavior and tunable mechanical properties in function of photo-initiators concentrations [118].

Temperature stimulus has also been considered for in situ gelling of polymers. Thermosensitive hydrogels for stem cell delivery are advantageous because their gelling process is triggered only by temperature variation, without using chemical crosslinkers that may raise cytotoxic phenomena [119]. An example of thermosensitive hydrogels is the one recently developed by Niu and co-workers. Their hydrogel solution, composed by N-isopropylacrylamide (NIPAAm), 2-hydroxyethyl methacrylate (HEMA), 1-vinyl-2-pyrrolidinone (VP), and acrylateoligolactide (AOLA), undergoes sol-gel transition when close to the room temperature. The latter was user-friendly to be injected at a temperature of 4 °C and it completely and quickly gelled at body temperature within 7 s reaching mechanical properties similar to those of heart tissue, due to its high flexibility. Moreover, the obtained hydrogels were photoluminescent, with a low photo-bleaching, that allowed to control the behavior of hydrogel after injection, in terms of degradation and release of the encapsulated cells, using a non-invasive tool. Through this method, the authors have shown that the degradation is a consequence of the hydrolysis of ester bonds, and that the production of acrylic acid unit from acrylate-oligolactide units occurred, so leading to the increase of hydrogel hydrophilicity and thermal transition temperature. Finally, the degradation products could dissolve in body fluids to be removed without toxicity [120]. At the same time, Ke et al., also developed another temperature-responsive system made of components with high biocompatibility and biodegradability (chitosan and β-glycerophosphate), loaded with HUVECs and NIH 3T3 cells. Authors proved that the addition of dextran in this formulation was useful to accelerate the gelation that occurred at 37 °C. Thanks to a concentration of 1% *w*/*v* of dextran, the in situ gelling process occurs in only 10 ± 1 s, more rapid if compared to that formulation without dextran (347 ± 6 s). Moreover, monitoring the expression of specific heart markers, they also tested and proved that cells were able to differentiate and to guarantee a therapeutic efficacy in cardiac tissue repair if delivered into the gel [121].

Finally, pH is another commonly used gelling stimuli for in situ-gelling hydrogel. As recently showed by Li and co-workers, the selective gelling in a specific range of pH could be advantageous to translate the use of injectable hydrogels in the clinical practice. Authors developed a propylacrilic acid (PAA)-based hydrogel, pH-sensitive and thermo-sensitive, that form gel at the pH of an infarcted heart (6–7) but not at a blood pH of 7.4 and this permitted to be administered without obstruction problems through catheters, that is the standard percutaneous invasive technique for cardiac interventions after MI. Cardiosphere-derived cells (CDCs) were also encapsulated in this hydrogel showing a good survival and differentiation [122].

These gelling characteristics, and the injectable hydrogels formation for cardiac tissue engineering, could be obtained exploiting several natural or synthetic polymers, many of which are the same of the ones used for the realization of cardiac patches. Natural biomaterials, such as dECM, harbour soft mechanical properties to be suitable for injection but they do not have good strength properties such as to guarantee the needed mechanical support for damaged heart tissue. If modified, dECM represents an excellent starting material to realize scaffold with kPa similar to that of the phatophysiological heart tissue [123]. For example, it has been demonstrated that the crosslinking of dECM, obtained from a porcine heart, with genipin alone or combined with different amount of chitosan, was able to improve the dECM mechanical properties to make it more suitable for heart application. Moreover, the addition of mesenchymal stem cells surprisingly demonstrated the gel remodeled to adapt to cell morphology ensuring their high viability and a better therapeutic functionality [124]. Fibrin has also been proposed as natural material for cardiac regeneration, due to its known role in hemostasis and tissue repair [125]. Particularly, it has been one of the first natural materials to be investigated for cellular cardiac administration, showing to improve post MI cardiac function due to its features of biocompatibility, biodegradability and angiogenesis induction. However, it has been found that its efficacy is limited over time [59]. Collagen is the main structural component of the animal extracellular matrix, that is able to guarantee the mechanical support and regulation of tissue activities. It could be extracted from several sources and purified to obtain a porous scaffold that is poorly immunogenic, biocompatible and biodegradable, all ideal features for tissue engineering. For these reasons, it has been proposed for this field in order to safely confer tissue support. However, the availability of collagen extracted from animals is limited in quantity and it may be necessary to use new materials that mimic the natural counterpart [126]. Alginate is another material that has been investigated for cardiac tissue engineering. It is noteworthy that the first injectable hydrogel made of acellular material to be tested in clinical trials was an alginate hydrogel. Alginate injectable hydrogel is contourable, therefore it adapts to the damaged tissue with whom it comes in contact and it is able to deliver cells in several tissue to induce repair [127]. Taking into consideration the advantages and limitations of these natural polymers, Montalbano and collaborators recently synthesized and investigated a hydrogel made of collagen, alginate and fibrin, using different collagen concentrations (0.5–2.5%), to mimic the external matrix employing a suitable cell delivery system to be used for soft tissue engineering. Authors performed in vitro studies, demonstrating that the obtained thermosensitive and porous scaffold had good cytocompatibility on several cell lines, including human mesenchymal stem cells, and it also showed similar mechanical properties compared to the native tissue, that was the main limit of natural materials [128]. However, in vivo studies are needed to test the efficacy of this hydrogel for tissue engineering.

Additionally, several synthetic materials [129,130,131] have been investigated to refine typical features of natural materials such as biochemical and mechanical properties, poor electrical conductivity and rapid degradation. Despite their ability to improve some aspects of the injectable hydrogel derived from natural polymers, synthetic hydrogels do not show the same high biocompatibility and intrinsic sites of interaction with cells that are found in natural materials. Moreover, the synthesis of suitable polymers often leads to a large consumption of solvents which is neither economic nor eco-sustainable. Both natural and synthetic polymers by themselves are not currently able to provide an ideal material and therefore a combination of them could be needed [123,132].

Despite the best material is still unknown, the use of natural and synthetic polymers or a combination of them to realize pre-formulations, able to respond to certain physical or chemical stimuli, still remains the best-proposed treatment for tissue engineering. The injectability of gelling biomaterial solution and its in situ sol-gel transition permit approaching application in clinical practice due to the required minimally invasive technique. Unfortunately, some aspects must be better defined and/or refined, such as the use of specific catheters able to guarantee a suitable application for programmed gelling. Ionic or pH-sensitive hydrogels, for example could require double channel injection catheters [123] to co-deliver the polymeric formulation and a fluid suitable to create the needed environment for triggering crosslinking reactions.

Moreover, the exclusive localization of the injected hydrogel in the infarcted cardiac area represents a hard task, due to the required time to complete jellification following a specific stimulus. In fact, if the jellification time is too long, the injected polymer solution could move from the application site following the currents of biological fluids before becoming a gel. For this reason, it is also important to better investigate the behavior of materials reaching incorrect sites.

Stimuli that induce jellification are key aspects to obtain a functional hydrogel, and the described studies and others have reached considerable successes, demonstrating that injectable hydrogels could satisfy some requirements of cardiac tissue regeneration, such as improvements in left ventricular remodeling, wall thickness and angiogenesis after MI, but still few stem cell-hydrogels have reached clinical practice. Further studies, for example, are needed to prove that delivered cells are able to survive, to migrate from and through the hydrogel, to adapt within the host tissue and differentiate in the cardiac environment in a minimal time from the injection for cardiac repair [133]. As already described for the patches, a limit to the pre-clinical studies is the use of animals with a cardiac organ very different from human. The efficacy proven in the rat and mouse animal models will not necessarily translate into similar beneficial results when applied to the humans, and further investigations on large animals more similar to humans, are needed to better define the exact dose of hydrogel to be administered and the right injection timing [123].

## 5. Conclusions and Future Perspectives

Through the use of preformed patches or injectable hydrogels, stem cell delivery certainly is set to improve the effectiveness of cell therapy treatments in regenerating and repairing the infarcted heart. Thanks to the aforementioned systems, the delivered cells are protected from the hostile cardiac microenvironment and their engraftment is promoted. Although pre-formed patches and injectable hydrogels have many common advantages, some limitations of their use have not yet been overcome. Consequent to the recent advances in tissue engineering, some problems such as poor vitality of encapsulated cells, frequent induced arrhythmias, the risk of rejection and poor implant efficiency, have been partially overcome by perfecting and functionalizing the biomaterials and improving the preparation methods. Despite this, the number of stem cell delivery systems that have reached clinical practice is still too low. A problem yet to be resolved, more closely related to patches, is the implantation method. To date, patches not only require an open chest surgery to be implanted in the specific cardiac site, but in some cases the application of biocompatible glues or sutures is necessary to ensure the maintenance of their position. In this scenario, while not eliminating the invasiveness problem of a real cardiac surgery, the functionalization of a patch with mucoadhesive molecules can certainly increase its stability on a continuously moving heart. Concurrently, it remains important to note that several patients, and diabetics in particular, require coronary interventions by standard cardiac surgery [5,134]. These patients often present with large scars that could be reached and treated only by applying a developed patch with multi lineage differentiated cells. On the other hand, injectable hydrogels are more easily administered with a minimally invasive technique, but their use in the clinic is not without difficulties yet to be investigated and overcome. In our opinion, the jellification time of the hydrogels, currently proposed as stem cell delivery systems, is still too long. Obtaining an injectable hydrogel with specific gelling properties able to undergo an almost instantaneous sol-gel transition could be the solution to any loss of biomaterial and encapsulated cells in the post-injection cardiac environment. Finally, the costs of engineered patches or hydrogels loaded with regenerative cells remains an issue for autologous cell therapy considering that it would constitute a highly personalized therapy to be available at each clinical site for delivery in patients. Costs will be still high if allogenic cells will be employed and therefore their costs will be acceptable only if their efficacy would be widely significant in the clinical scenario.

Certainly, tissue engineering with its recent advances is on the right path to find a therapeutic solution that will lead to complete resolution of heart damage, but further efforts still need to be made to efficiently take these scaffolds from bench to bedside.

## Figures and Tables

**Table 1:** A comparison of the most common tissue engineering approaches.

Approaches	Advantages	Limitations	Studies
Cell/stem cell therapy	Minimally invasive strategies (catheter or endocardial-based injection)	Landing in hostile environment and low cell survival	[58,59,60,62,63]
		Inability to immediately act	
		Low engraftment	
		Low conductive stimuli for cells	
		Mechanical loss due to squeezing out from myocardium	
Patch-assisted cell delivery	Specific myocardial injection	Open chest surgery required with low patient compliance	[64,65,66,67]
	Increased cell retention and engraftment	Thickness is limiting for cellular mobility	
	Technological-formulative versatility	Low adaptability with host tissue	
	Protection of cells from hostile post-MI microenvironment	Arrhythmia and immunological response induced	
		Necessary integration of nutrients and biomolecules inducing cell viability	
	Protection of cells from post-MI microenvironment	Fastening system often required	
		Clinical translation currently limited	
Injectable hydrogel-assisted cell delivery	Minimally invasive administration	Necessary integration of nutrients and biomolecules inducing cell viability	[64,68,69,70]
	Increased cell retention and engraftment	Poor electrical connection with the host	
	Technological-formulative versatility		
	Protection of cells from hostile post-MI microenvironment	Clinical translation currently limited	
	Protection of cells from post-MI microenvironment	Not instantaneous in situ gelling	

## Abbreviations

CVD	Cardiovascular disease
CSC	Cardiac stem cell
MI	Myocardial infarction
CHF	Chronic heart failure
ACE	Angiotensin-converting enzyme
CM	Cardiomyocytes
dECM	Decellularized extracellular matrix
PGS	Poly(glycerol sebacate)
hESC-CM	Human embryonic stem cell-derived cardiomyocytes
VEGF	Vascular endothelial growth factor
